# Pet ownership and maintenance of cognitive function in community-residing older adults: evidence from the Baltimore Longitudinal Study of Aging (BLSA)

**DOI:** 10.1038/s41598-023-41813-y

**Published:** 2023-09-07

**Authors:** Erika Friedmann, Nancy R. Gee, Eleanor M. Simonsick, Melissa H. Kitner-Triolo, Barbara Resnick, Ikmat Adesanya, Lincy Koodaly, Merve Gurlu

**Affiliations:** 1 Department of Organizational Systems and Adult Health, University of Maryland School of Nursing, 655 W. Lombard St., Suite 402, Baltimore, MD 21201 USA; 2 Department of Psychiatry, Center for Human Animal Interaction, School of Medicine, Virginia Commonwealth University, Richmond, VA USA; 3 Intramural Research Program, National Institute on Aging, National Institutes of Health, Baltimore, MD USA

**Keywords:** Psychology, Diseases, Medical research

## Abstract

Pet ownership has been associated with reduced deterioration in physical health as older adults age; little research focused on deterioration in cognitive function. We examine the relationship of pet, dog, cat ownership, and dog walking to changes in cognitive function among 637 generally healthy community-dwelling older adults (185 pet owners) aged 50–100 years (M = 68.3, SD = 9.6) within the BLSA. Cognitive assessments every 1–4 years over 1–13 years (M = 7.5, SD = 3.6) include the California Verbal Learning (Immediate, Short, Long Recall); Benton Visual Retention; Trail-Making (Trails A, B, B-A); Digit Span; Boston Naming (Naming); and Digit Symbol Substitution (Digit Symbol) Tests. In linear mixed models, deterioration in cognitive function with age was slower for pet owners than non-owners (Immediate, Short, Long Recall; Trails A,B,B-A; Naming; Digit Symbol); dog owners than non-owners (Immediate, Short Recall; Trails A,B; Naming; Digit Symbol); and cat owners than non-owners (Immediate, Short, Long Recall; Naming), controlling for age and comorbidities. Among dog owners (N = 73) walkers experienced slower deterioration than non-walkers (Trails B, B-A; Short Recall). All ps ≤ 0.05. We provide important longitudinal evidence that pet ownership and dog walking contribute to maintaining cognitive function with aging and the need to support pet ownership and dog walking in design of senior communities and services.

## Introduction

Memory^1–5^ and selected cognitive functions^2,3^ decline as adults age with steeper declines in advanced age^2,4^, even in the absence of dementia. As the proportion of older age cohorts increase over the next few decades the need to address care and health needs of older adults will increase^6^. Promotion of successful aging is intended to reduce the current five to seven-year gap between high quality of life and overall life expectancy. to enable people to function their best for as long as possible^7^. Many strategies can help minimize the cognitive decline including treating sleep apnea, improving diet, and increasing exercise^8^. The current study examines the potential of pet ownership as a strategy to slow the decline in cognitive function with aging in generally healthy community-residing older adults.

The biopsychosocial model can be applied to explain the potential contribution of pet ownership to health and successful aging^9,10^. In this model, health is viewed as a continuous outcome, influenced by challenges to and promoters of the biological, social, and psychological realms. Each realm impacts the others, with all three combining dynamically over personal and historical time to construct overall individual health.

Human-animal interaction and pet ownership commonly are conceptualized as components of the social realm. Social support from a pet can impact other components within the social realm, as well as those in the biological and psychological realms^11^. Influence of pets on the biological realm can include decreases in biological stress indicators such as cortisol, blood pressure, and heart rate^10^. The biological and psychological realm impacts often are closely intertwined. for example, interacting with a dog can decrease anxiety, reflected by changes in stress biomarkers. Interacting with a companion animal can provide social support as well as a source of bonding and attachment all of which promote better psychological health.

Research suggests that by providing social support, pet ownership has the potential to contribute to the maintenance of cognitive function within the psychological realm and thus promote successful aging^9,12^. Research supports that among older adults human–animal interaction can provide social support, increase social interaction, and decrease loneliness^13^, which are associated with maintaining cognitive function^14^. The differences in social isolation and loneliness among older adults according to pet ownership largely occur in socially isolated individuals^15^. Socially isolated older adults often turn to their pets as sympathetic ears^16^. Ample evidence supports the notion that the presence of an animal encourages social interaction. For example, The presence of a dog catalyzed social interactions by increasing approaches by strangers, even those who appeared unkempt^17^. Even in individuals with dementia, visits from a friendly dog improved both the quantity and the quality of social interaction in residents of care facilities^18^.

Social relationships have been related to slower cognitive decline among older adults^14^. Research examining the relationship of human-animal interaction to human cognition suggests that human-animal interaction may enhance cognitive functioning by reducing stress. Improvements in executive function for university students at-risk of academic failure occurred when the students had multiple opportunities to interact with dogs compared with when they had access to formalized academic stress management content^19^. Both animal assisted and yoga interventions reduced children’s stress similarly and improved their spatial abilities more than a control condition^20^. A number of other studies with small sample sizes also demonstrated improvements in aspects of cognition when individuals, including older adults, interacted with dogs^21^.

A few cross-sectional studies directly examine the relationship of pet ownership to cognitive function in older adults. In a community sample of older adults in the Health and Retirement Study, individuals aged 65 and older who had owned pets for more than five years had better overall cognition and verbal memory than individuals who did not own pets^22^. In a small sample (N = 88) of community-residing homebound older adults pet owners had better executive function than non-owners and executive function did not differ between dog and cat owners in unadjusted analysis^23^. In a similarly sized study of community residing older women, the majority of whom exhibited mild cognitive impairment, cat ownership was not related to executive function without adjustment for other demographic variables^24^. In contrast, among a larger group of healthy community-residing older adults, pet ownership and dog ownership within the past ten years were related to better verbal learning, memory and visual perception after controlling for age^25^.

Dog and cat owners interact differently with their pets which might be associated with differences in ageing-related changes in cognitive function. A recent longitudinal study examined cat ownership in relation to changes in cognitive status from unimpaired to impaired in community-residing older adults. Cat ownership was not related to the development of mild cognitive impairment after adjustment for potential confounders^26^.

In addition to examining the contribution of overall pet ownership, the current study examines the independent associations of dog ownership and cat ownership with changes in cognitive function in generally healthy community-dwelling older adults. Few studies investigated the differential contributions of ownership of individual pet species to cognitive status. Most intervention studies that demonstrate effects on cognitive status evaluated only dog-based interventions^21^.

Owning pets, especially dogs has been related to physical activity, which is expected to support health. Data from the Health ABC Study and the Health and Retirement Study (HRS) support these relationships. Older adult dog owners exercised more than non-owners^27–29^. Compared with older adults who don’t own pets, dog walkers had lower body mass, said they could complete more activities of daily living, and made fewer visits to health care professionals^27^.

The current study is the first longitudinal examination of the relationship of dog walking to changes in cognitive function. In a national epidemiological cohort study of older adults that controlled for confounders, more baseline physical activity was related to better maintenance of executive function, memory, and semantic and letter fluency over an average of 3 years. Baseline physical activity was also related to lower odds of developing cognitive impairment after controlling for potential confounders^30^. A large body of research supports the benefits of exercise programs for maintaining cognition in older adults with cognitive impairment^31^ or specific illnesses^32^. For example, in a randomized controlled trial for individuals whose cognition was mildly impaired due to multiple sclerosis, cognition (symbol digit modalities test) improved with an exercise program^33^. We expect that dog walking will be related to slower deterioration in cognition with aging in this population.

The current study examines the relationship of pet ownership to incremental changes in cognitive function over 1 to 13 years (M = 7.5, SD = 3.6) in generally healthy community-dwelling older adults. We asked:

1. Is pet ownership related to slower deterioration of cognitive function with aging?
2. Is dog ownership or cat ownership related to slower deterioration of cognitive function with aging?
3. Is dog walking related to slower deterioration of cognitive function with aging?
4. Is cognitive status related to deterioration in cognitive function with aging?

## Methods

### Design

This cohort study used data collected from the BLSA, the United States’ longest-running scientific study of human aging, initiated in 1958. The BLSA addresses crucial questions about the normal and pathological age-related changes using a longitudinal observational design. At each 3-day visit from March 2017 to March 2020 to the BLSA participants completed a standardized battery of cognitive assessments. Frequency of BLSA visits increases with aging and ranges from every 4 years for the youngest participants to annually for those aged 80 years and older. The BLSA study and the addition of the human-animal interaction questionnaire were approved by the National Institutes of Health Intramural Research Program Institutional Review Board (IRB). All participants provided written informed consent prior to participation. Study procedures were conducted in accordance with the Helsinki Agreement and all relevant US human subjects’ guidelines and regulations.

The current study uses data from participants who were aged 50 years and above when they completed their first human-animal interaction assessment (index visit) and who had completed at least one BLSA assessment in the previous ten years or completed a second BLSA assessment prior to March 2020. The index visit data includes both pet ownership (retrospective) and BLSA (contemporaneous) cognitive function up to ten years before the survey visit. Time from the index visit to the survey visit ranged from one to ten years during which the participants completed BLSA visits at regularly scheduled intervals. Participants also performed cognitive function assessments during their regularly scheduled BLSA visits for 3 years after the survey visit. Thus, participants’ cognitive function data were available for from one to 13 years. Cognitive data from the index visit serve as the baseline for examination of changes in cognitive function with aging.

## Measures

### Pet ownership measures

Multiple sets of questions elicited information about pet ownership including a ten-year pet ownership history questionnaire and dog walking behavior from the pet ownership module of the Health and Retirement Study (HRS)^34,35^. These questions were administered during BLSA visits from March 2017 through March 2020. Questions for pet ownership “Do you currently have any pets?” and “What kind of pets are these?” Separate questions asked numbers of each species of pets, with species listed including cats, dogs, birds, small mammals, fish, reptiles, and others. To obtain pet ownership history for ten years prior to the survey visit, participants were asked to complete a grid that requested they check “What type of pet was owned “1 year ago, 2 years ago, 3 years ago,” through 10 years ago. For each year participants were asked to check “yes” or “no” for specific species including cat, dog, bird, and other animal. The dog walking behavior question used in this study was “Do you walk your dog?”.

### Cognitive function measures

The assessment uses a standardized neurocognitive battery of reliable and well validated tests that are sensitive to small changes in cognitive function. The BLSA cognitive assessment includes tests of several large cognitive function domains including executive function and language function as well as more specific domains of executive function: processing speed, verbal, non-verbal, and working memory, and attention.

The California Verbal Learning Test^36^ is a test of verbal learning and memory. Participants were read 16 shopping items, four from each of 4 sematic categories, over five learning trials. Three measures were derived and used in this study: Immediate Recall (total number of items recalled across the five learning trials); short-delay free recall (Short Recall: number of items recalled immediately after being read an interference shopping list), and long-delay free recall (Long Recall: number of items recalled after a 20-min delay). Higher scores indicate better recall.

The Benton Visual Retention Test (Visual Recall)^37^ is a measure of short-term non-verbal (figural) memory and visuospatial ability. Participants studied line drawings (designs) of one to three geometric figures, for ten seconds each. They then immediately drew the design from memory. The designs become more difficult over the ten designs. The total number of errors provided the score for Visual Recall. Lower scores indicate better performance.

Executive function refers to a set of cognitive control processes that facilitate goal-directed behavior and are considered to be essential to navigating nearly all aspects of human life including occupational and career success, interpersonal relationships, physical and mental health, and day-to-day functioning^38^. Psychomotor speed and processing speed are domains within executive function. Psychomotor speed is required to manipulate and/or maintain information over brief periods of time and to allocate attentional resources as needed to various tasks^39^. Processing Speed is the time it takes for a person to perform a mental task. Trail Making A (Trails A) and B (Trails B)^40^ are tests of perceptuomotor speed, visuomotor scanning (Trails A and Trails B), attention (Trails A), and concentration and set shifting (Trails B). In Trails A participants drew a line to connect randomly arranged numbers from 1 to 25 in sequential order. In Trails B participants drew a line to connect alternate randomly arranged numbers 1–13 and letters A-L in sequential order (e.g., 1-A-2-B...). Participants were asked to be as quick and accurate as possible. Scores represent the number of seconds it took for completion; lower scores indicate better performance. A lower difference between the two scores (Trails B-A) also indicates better performance.

Digit Span Test (Digits) Forward (Digits Forward) and Backward (Digits Backward)^41^ are measures of short-term memory span (Digits Forward) and executive function, specifically working memory (Digits Backward). With Digits Forward, participants were read increasingly longer lists of digits which they repeated in forward order. With Digits Backward participants were read increasingly longer lists of digits which they repeated in reverse order. The scores for forward and backward were the total scores with a maximum of 14 in each direction. Higher scores indicate better performance.

Digit Symbol Substitution Test (Digit Symbol)^41^ is a measure of psychomotor speed, executive function and visual-perceptual integration. Participants were given a code sheet with numbers from 1 to 9 with symbols matched to each. They used the codes to insert the symbols below each number. The number of correct symbols placed below the numbers within 90 s provides the score with higher scores indicating better performance.

The Boston Naming Test (Naming)^42^ is a measure of language function including confrontational naming and semantic recall. Participants were asked to identify and name a series of 60-line drawings of objects. The drawings begin with common objects and end with infrequent ones. Incorrect answers were cued by providing a stimulus cue for perceptual errors and a phonemic cue for semantic errors. The score on the test was the number of items identified correctly without cues, thus higher scores indicate better naming.

### Covariates

Aging and comorbidities are typically associated with decreasing or impaired cognitive function^43^. These variables were chosen a priori as potential confounders to the relationship between pet ownership and cognitive function as pet owners were younger and had fewer comorbidities than non-owners. Comorbidity scores represent how many of eight conditions [heart disease (including angina pectoris, myocardial infarction, heart failure, angioplasty, coronary artery bypass graft), diabetes, pulmonary disease, cerebral vascular disease, lower extremity arthritis, lower extremity pain, minor functional difficulty, and exertional pain while walking] the participant affirmed experiencing^25^.

### Cognitive impairment

An 11 item mini mental state examination (MMSE) was used to assess overall cognitive status at each study visit^44^. Scores ≤ 24 are generally considered to indicate a degree of cognitive impaired^45^.

### Statistics

Descriptive statistics were used to portray the participants and their baseline characteristics. Characteristics of pet owners and non-owners at initial human-animal interaction assessment within the BLSA and at the first time for which both pet ownership and BLSA functional data are available were compared using t-tests for normally distributed continuous variables, Wilcoxon rank sum test for non-normally distributed continuous variables, and chi square tests for categorical variables. Differences in baseline cognitive function between cat and dog owners and dog owners who walked and did not walk their dogs were examined similarly.

Changes in cognitive function according to pet ownership could be examined beginning with contemporaneously recorded historic records of cognitive assessments in the BLSA within the ten-year period covered by the retrospective pet ownership history section of the human-animal interaction assessment. Pet ownership history was matched with cognitive assessments for the ten years prior to the BLSA survey visit and with simultaneous human-animal interaction and cognitive function assessments from March of 2017 through March of 2020. Pet ownership history follow-up ranged from ten to 12.6 years zero.

Prior to multivariable analysis, data were cleaned and examined for outliers and normality. Trails B was natural log transformed, Trails A and Trails B-A were Winsorized at a high of 150 s and a low of 0 s, respectively, and natural log transformed, Naming was reflected by subtracting raw scores from 61 (maximum score plus one) and then natural log transformed to attain normality. Intraclass correlations indicated considerable dependence ranging from 0.51 (Trails B-A) to 0.88 (Naming; Supplemental Table 3).

Linear mixed models (LMMs) with random intercepts and repeated measures for participants were used to examine changes in cognitive outcomes with aging and to compare the changes according to pet ownership status. Pet ownership status was included as a time varying predictor by associating status at the time of each cognitive assessment with similarly timed outcome measures over up to 13 years [mean (M) = 7.5, standard deviation (SD) = 3.59]. Age and comorbidities were included as covariates in all LMM analyses. Separate LMMs were used to examine the association of pet ownership, dog ownership, and cat ownership with changes in each cognitive function outcome with aging. LMMs that simultaneously included cat ownership and dog ownership were used to examine the independent associations of cat and dog ownership to longitudinal changes in each cognitive function outcome. A third series of LMMS was used to compare the associations of cat ownership versus dog ownership with longitudinal changes in cognitive function outcomes. Similar LMMs were employed to examine the relationship of dog walking to overall cognitive function outcomes and changes in the cognitive function outcomes. Lastly, LLMs examined differences in changes in cognitive function outcomes according to pet ownership between those who were and were not cognitively intact.

We calculated Cohen’s d effect sizes (ES) for the significant interactions of aging and pet ownership status. We calculated the difference in change in outcomes over ten years between pet owners and non-owners and divided this by the raw baseline outcome standard deviation. For transformed variables, the standard deviation (SD) of the transformed variable was used in the calculation. Analyses were conducted with SPSS 28 (IBM, Armonk NY) and Stata SE 16 (College Station, Tx).

## Results

A total of 637 BLSA participants met the inclusion criteria. Ages when they at the survey visit ranged from 50.8 to 100.80 years (mean = 75.09 years, SD = 10.15). Most participants were White (66.98%) with smaller percentages of Blacks (28.12%), Asians (1.26%), and Hawaiian/Pacific Islanders (0.32%); 53.89% were women. Participants were highly educated; most (67.03%) held a postgraduate degree. The sample predominantly was married (61.89%), lived with at least one other person (56.62%), resided in a single-family house (78.74%), and had an annual income greater than $50,000 (83.39%). Of the 637 participants at the survey visit 185 (29.0%) kept pets; 67 (10.52%) kept cats, and 84 (13.19%) kept dogs, with few people keeping other animals. Most of the dog owners (69.05%) stated that they walked their dogs.

At the index visit (Table 1), participants were generally in good health with a mean of 0.96 (SD = 1.17) comorbidities; and only a small percentage of the entire sample (1.3%, N = 8) had MMSE scores indicating the possibility of some degree of cognitive impairment. One hundred eighty-eight (29.51%) of the 637 participants kept pets; 67 (10.52%) kept cats and 84 (13.19%) kept dogs; few people kept other animals. Among the dog owners, 58 (79.5%) reported walking their dogs. Pet owners were significantly younger and had fewer comorbidities than non-owners (Table 1). Pet owners were less likely than now-owners to be Black (15.96% vs 32.96%) and more likely to married (76.47% vs 63.53%), to live with one or more others (81.18% vs 71.3%), to work (60.43% vs 49.66%), and to live in a single-family home (95.16% vs 77.85%). Notably, 8 people lived in foster or assisted living communities and none of them kept pets. MMSE scores did not differ significantly according to pet ownership status.

**Table 1:** Demographic and pet ownership characteristics of respondents at the time of the BLSA index visit (follow-up = 0 years).

Characteristic	Overall (n = 637)		Pet non-owner (n = 449)		Pet owner (n = 188)		Value	*p*
	N or M	(%) or (SD)	N or M	(%) or (SD)	N or M	(%) or (SD)		
Dog owner, N (%)	84	(13.19)	0	n/a	84	(44.68)		
Cat owner, N (%)	67	(10.52)	0	n/a	67	(35.64)		
Age in years (yrs.), M (SD)^3^	68.25	(9.64)	69.47	(9.67)	65.33	(8.97)	5.03	** < 0.001**
Black, N (%)^1^	178	(27.94)	148	(32.96)	30	(15.96)	19.03	** < 0.001**
Female, N (%)^1^	281	(44.11)	200	(44.54)	81	(43.09)	0.11	0.735
< College Graduate, N (%)^1^	80	(12.62)	66	(14.47)	14	(7.49)	6.40	**0.042**
Married or partnered, N (%)^1^	427	(67.35)	284	(63.53)	143	(76.47)	10.03	**0.002**
Lives alone, N (%)^1^	163	(25.79)	128	(28.70)	35	(18.82)	6.70	**0.009**
Single family housing, N (%)^1^	525	(82.94)	348	(77.85)	177	(95.16)	25.33	** < 0.001**
Family income exceeds 50 K, N (%)^1^	508	(81.94)	349	(80.41)	159	(85.48)	2.32	0.127
Currently works, N (%)^1^	335	(52.84)	222	(49.66)	113	(60.43)	6.00	**0.014**
Currently volunteers, N (%)^1^	372	(58.68)	263	(58.84)	109	(58.29)	0.001	0.991
MMSE, M (SD)^3^	28.72	(1.43)	28.64	(1.29)	28.87	(1.69)	1.06	0.292
Comorbidities, M (SD)^4^	0.95	(1.17)	0.99	(1.15)	0.85	(1.22)		**0.030**
Immediate recall, M (SD)^3^	52.65	(11.53)	51.96	(11.77)	54.34	(10.75)	− 2.35	**0.019**
Short recall, M (SD)^3^	10.51	(3.33)	10.29	(3.35)	11.05	(3.20)	− 2.60	**0.010**
Long recall, M (SD)^3^	11.17	(3.28)	10.93	(3.30)	11.75	(3.15)	− 2.85	**0.005**
Visual recall, M (SD)^3^	6.85	(4.81)	7.15	(4.94)	6.12	(4.38)	2.44	**0.014**
Trails A(wln), M (SD)^3^	3.35	(0.32)	3.37	(0.33)	3.31	(0.30)	2.27	**0.023**
Trails B(ln), M (SD)^3^	4.26	(0.41)	4.28	(0.43)	4.22	(0.36)	1.77	0.077
Trails B-A(wln), M (SD)^3^	4.48	(0.35)	4.49	(0.38)	4.46	(0.27)	0.96	0.336
Digits forward, M (SD)^3^	6.68	(1.26)	6.60	(1.27)	6.84	(1.22)	− 2.17	**0.031**
Digits backward, M (SD)^3^	5.11	(1.32)	5.05	(1.32)	5.28	(1.31)	− 1.97	**0.049**
Naming(rln)*, M (SD)^3^	2.76	(0.29)	2.78	(0.31)	2.69	(0.24)	2.60	**0.010**
Digit symbol, M (SD)^3^	47.51	(11.18)	46.86	(11.25)	49.05	(10.86)	− 2.28	0.023

ln: natural log transformed; rln: reflected then natural log transformed; wln: Winsorized then natural log transformed; Immediate Recall: California Verbal Learning Test-Immediate Recall; Short Recall : California Verbal Learning Test-short delay free recall; Long Recall: California Verbal Learning Test-long delay free recall; Visual Recall: Benton Visual Retention Test; Trails A: Trail Making Test-A; Trails B: Trail Making Test-B; Trails B-A : Trail Making Test B-Trail Making Test A; Digits Forward: WAIS-R Digits Span Test-maximum Digits Forward; Digits Backward: WAIS-R Digits Span Test-maximum Digits Backward; Digit Symbol: WAIS-R Digit Substitution Test; Naming: Boston Naming Test Score.

^1^Chi-Square test; ^2^Fischer’s exact test; ^3^Student’s t-test; ^4^Wilcoxon rank sum test.

*Naming was reflected so higher scores indicate worse function.

That bold indicates statistical significance p < .05 is appropriate.

At the index visit, unadjusted cognitive function measure scores differed between pet owners and non-owners. Immediate Recall, Short Recall, Long Recall, Digits Forward, Digits Backward, and Digit Symbol were higher and Visual Recall (number of errors), Trails A (seconds to complete), and Naming (number correct, reflected prior to normalization) were lower for pet owners than non-owners (Table 1), indicating better performance on these measures for pet owners. Trails B and Trails B-A did not differ.

At the index visit assessment, individuals who owned dogs but not cats (dog owners exclusively) and individuals who owned cats but not dogs (cat owners exclusively) shared similar demographic characteristics (Table 2). Cat owners exclusively had significantly more comorbidities (M = 1.05, SD = 1.25; M = 0.75, SD = 1.15) and had been in the study for a longer time (M = 7.71, years SD = 3.38; M = 6.16 years, SD = 4.29) than dog owners exclusively. Index visit cognitive function variables did not differ significantly between cat owners exclusively and dog owners exclusively.

**Table 2:** Demographic and pet ownership characteristics of pet owners comparing those who own cats exclusively (without owning dogs) to those who own dogs exclusively (without owning cats) at index visit.

Characteristic	Overall (n = 141)		Cat owners n = 62)		Dog owners(n = 79)		Value	*p*
	N or M	(%) or (SD)	N or M	(%) or (SD)	N or M	(%) or (SD)		
Age in years (yrs.), M (SD)^3^	65.99	(9.22)	66.60	(8.58)	65.52	(9.72)	− 0.69	0.489
Black, N (%)^1^	26	(18.44)	10	(16.13)	16	(20.25)	0.39	0.531
Female, N (%)^1^	77	(54.61)	37	(59.68)	40	(50.63)	1.15	0.284
< College graduate, N (%)^2^	8	(5.71)	4	(6.45)	4	(5.13)		0.441
Married or partnered, N (%)^1^	108	(77.14)	49	(79.03)	59	(75.64)	0.23	0.635
Number of others in household, N (%)^1^	25	(17.86)	9	(14.52)	16	(20.51)	4.41	0.221
Single family housing, N (%)^2^	132	(94.96)	60	(98.36)	72	(92.31)		0.135
Family income exceeds 50 K, N (%)^1^	120	(86.96)	53	(86.89)	67	(87.01)	0.001	0.982
Currently works, N (%)^1^	84	(60.00)	35	(56.45)	49	(62.82)	0.58	0.445
Currently volunteers, N (%)^1^	79	(57.25)	39	(63.93)	40	(51.95)	2.00	0.158
MMSE, M (SD)^3^	28.96	(1.73)	29.39	(0.66)	28.65	(2.17)	1.59	0.117
Comorbidities, M (SD)^4^	0.8	(1.20)	1.05	(1.25)	0.75	(1.15)		**0.041**
Follow-up yrs., M (SD)^3^	6.84	(3.98)	7.71	(3.38)	6.16	(4.29)	− 2.33	**0.021**
Immediate Recall, M (SD)^3^	54.43	(10.96)	55.66	(11.12)	53.47	(10.81)	− 1.15	0.252
Short Recall, M (SD)^3^	10.93	(3.27)	11.34	(3.08)	10.61	(3.39)	− 1.30	0.197
Long Recall, M (SD)^3^	11.64	(3.28)	12.05	(2.90)	11.32	(3.54)	− 1.29	0.198
Visual Recall, M (SD)^3^	6.18	(4.59)	5.48	(4.26)	6.75	(4.79)	1.62	0.108
Trails A(wln), M (SD)^3^	3.34	(0.30)	3.33	(0.28)	3.34	(0.33)	0.10	0.924
Trails B(ln), M (SD)^3^	4.25	(0.38)	4.21	(0.34)	4.28	(0.41)	1.21	0.229
Trails B-A(wln), M (SD)^3^	4.48	(0.30)	4.43	(0.31)	4.51	(0.29)	1.55	0.123
Digits forward, M (SD)^3^	6.90	(1.22)	7.05	(1.17)	6.79	(1.27)	− 1.25	0.214
Digits backward, M (SD)^3^	5.30	(1.31)	5.38	(1.14)	5.24	(1.43)	− 0.61	0.545
Naming(rln)*, M (SD)^3^	2.71	(0.24)	2.69	(0.25)	2.72	(0.24)	0.53	0.596
Digit symbol, M (SD)^3^	48.15	(11.31)	48.77	(11.44)	47.63	(11.25)	− 0.59	0.557

ln: natural log transformed; rln: reflected then natural log transformed; wln: Winsorized then natural log transformed; Immediate Recall: California Verbal Learning Test-Immediate Recall; Short Recall: California Verbal Learning Test-short delay free recall; Long Recall: California Verbal Learning Test-long delay free recall; Visual Recall: Benton Visual Retention Test; Trails A: Trail Making Test-A; Trails B: Trail Making Test-B; Trails B-A: Trail Making Test B-Trail Making Test A; Digits Forward: WAIS-R Digits Span Test-maximum Digits Forward; Digits Backward: WAIS-R Digits Span Test-maximum Digits Backward; Digit Symbol: WAIS-R Digit Substitution Test; Naming: Boston Naming Test Score; Digit Symbol: WAIS-R Digit Substitution Test.

^1^Chi-Square test; ^2^Fischer’s exact test; ^3^Student’s t-test; ^4^Wilcoxon rank sum test.

*Naming was reflected so higher scores indicate worse function.

That bold indicates statistical significance p < .05 is appropriate.

### Changes in cognitive function with aging

**Figure 1:**
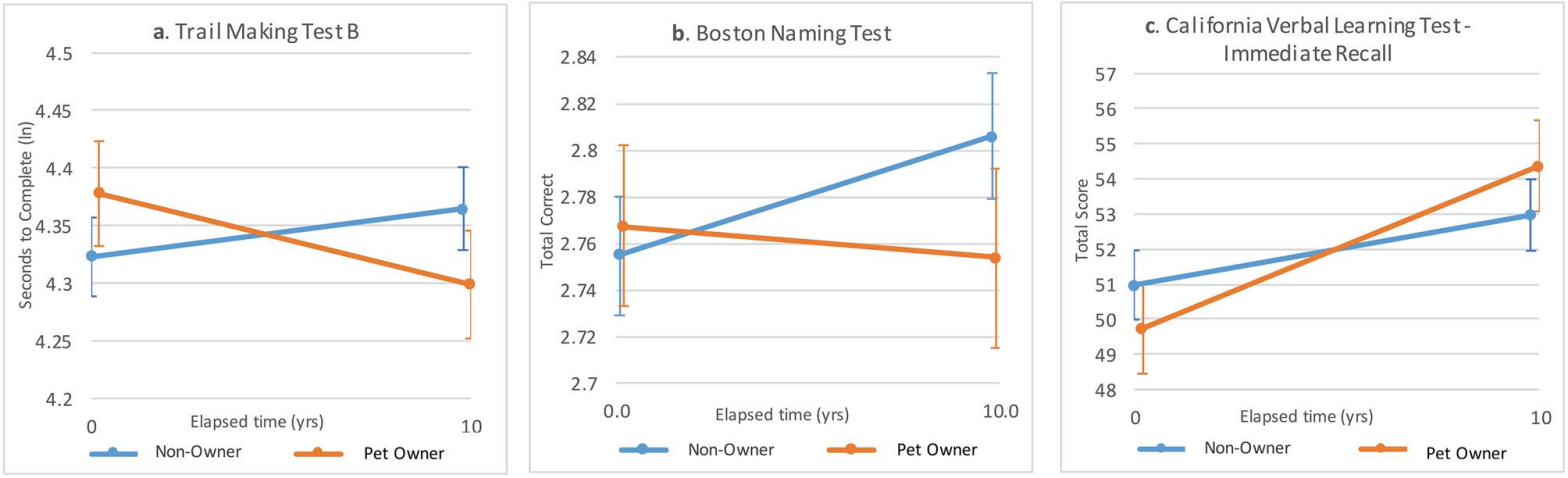
Changes in selected measures of cognitive function with aging: comparison of pet owners and non-owners. Note (ln) in axis label indicates a natural log transformation was applied.

All cognitive function outcomes in unadjusted analyses deteriorated significantly as participants aged (Table 3). Pet ownership moderated the changes in cognitive function as participants aged after controlling for age and comorbidities (Table 4). Pet owners experienced significantly different trajectories of change than non-owners with pet owners demonstrating less deterioration in Immediate Recall (ES = 0.23), Short Recall (ES = 0.15), Long Recall (ES = 0.13), Trails A(ES = − 0.25), Trails B (ES = − 0.29), Trails B-A (ES = − 0.14), Naming (ES = 0.21) and Digit Symbol (ES = 0.14) compared to non-0wners (see Fig. 1).

**Table 3:** Outcome of bivariate linear mixed models showing changes in cognitive function as individuals aged (N = 637).

Outcome	Estimate	se	*p*
Immediate recall	− 0.192	0.030	< 0.001
Short recall	− 0.083	0.009	< 0.001
Long recall	− 0.085	0.009	< 0.001
Visual recall	0.604	0.014	< 0.001
Trails A(wln)	0.014	0.001	< 0.001
Trails B(ln)	0.017	0.001	< 0.001
Trails B-A(wln)	0.012	0.001	< 0.001
Digits forward	− 0.025	0.004	< 0.001
Digits backward	− 0.026	0.004	< 0.001
Naming(rln)*	0.004	0.001	< 0.001
Digit symbol	− 1.103	0.022	< 0.001

ln: natural log transformed, rln: reflected then natural log transformed, wln: winsorized then natural log transformed, Immediate Recall: California Verbal Learning Test-Immediate Recall, Short Recall: California Verbal Learning Test-short delay free recall, Long Recall: California Verbal Learning Test-long delay free recall, Visual Recall: Benton Visual Retention Test, Trails A: Trail Making Test-A, Trails B: Trail Making Test-B, Trails B-A: Trail Making Test B-Trail Making Test A, Digits Forward: WAIS-R Digits Span Test-maximum Digits Forward, Digits Backward: WAIS-R Digits Span Test-maximum Digits Backward, Digit Symbol: WAIS-R Digit Substitution Test, Naming: Boston Naming Test Score.

*Naming was reflected so higher scores indicate worse function.

**Table 4:** Changes in cognitive function variables with aging according to pet ownership, dog ownership, and cat ownership, adjusted for age and comorbidities (n = 637).

Outcome	Pet ownership interaction with years of aging			Dog ownership interaction with years of aging			Cat ownership interaction with years of aging		
	Est	se	*p*	Est	se	*p*	Est	se	*p*
Immediate recall	0.264	0.071	** < 0.001**	0.239	0.102	**0.019**	0.231	0.099	**0.021**
Short recall	0.051	0.021	**0.018**	0.060	0.031	**0.049**	0.061	0.030	**0.042**
Long recall	0.043	0.021	**0.040**	0.043	0.030	0.151	0.071	0.029	**0.014**
Visual recall	− 0.002	0.032	0.959	0.098	0.047	**0.035**	− 0.058	0.045	0.199
Trails A(wln)	− 0.008	0.002	** < 0.001**	− 0.007	0.003	**0.025**	− 0.004	0.003	0.185
Trails B(ln)	− 0.012	0.003	** < 0.001**	− 0.010	0.004	**0.009**	− 0.005	0.004	0.155
Trails B-A(wln)	− 0.005	0.003	**0.042**	− 0.007	0.004	0.072	− 0.001	0.004	0.763
Digits forward	0.010	0.009	0.269	− 0.002	0.013	0.871	0.002	0.013	0.878
Digits Backward	0.017	0.010	0.072	0.018	0.014	0.178	− 0.010	0.013	0.466
Naming(rln)*	− 0.006	0.001	** < 0.001**	− 0.005	0.002	**0.024**	− 0.005	0.002	**0.016**
Digit symbol	− 0.155	0.050	0.002	− 0.172	0.073	0.018	− 0.082	0.070	0.243

Reference category is not-ownership.

ln: natural log transformed; rln: reflected then natural log transformed; wln: Winsorized then natural log transformed; Immediate Recall: California Verbal Learning Test-Immediate Recall; Short Recall: California Verbal Learning Test-short delay free recall; Long Recall: California Verbal Learning Test-long delay free recall; Visual Recall: Benton Visual Retention Test; Trails A: Trail Making Test-A; Trails B: Trail Making Test-B; Trails B-A: Trail Making Test B-Trail Making Test A; Digits Forward: WAIS-R Digits Span Test-maximum Digits Forward; Digits Backward: WAIS-R Digits Span Test-maximum Digits Backward; Digit Symbol: WAIS-R Digit Substitution Test; Naming: Boston Naming Test Score.

*Naming was reflected so higher scores indicate worse function.

That bold indicates statistical significance p < .05 is appropriate.

### Dog ownership, cat ownership, and changes in cognitive function

In separate analyses controlling for age and comorbidities, both dog ownership and cat ownership were related to changes in cognitive function (Table 4). Dog owners experienced significantly less deterioration in Immediate Recall (*p* = 0.019, ES = 0.21), Short Recall (*p* = 0.049, ES = 0.13), Trails A (*p* = 0.025, ES = − 0.22), and Trails B (*p* = 0.009, ES = − 0.24), than people who did not own dogs. Dog owners experienced significantly different trajectories of Naming (*p* = 0.024, ES = 0.17), and Digit Symbol (*p* = 0.018, ES = 0.15) than non-owners; owners’ scores increased (improved) while non-owner’ scores decreased (deteriorated). Cat owners exhibited significantly different trajectories in Immediate Recall (*p* = 0.021, ES = 0.20) and Naming (*p* = 0.016, ES = 0.17) than individuals who did not own cats, with cat owner scores improving and non-owners scores deteriorating. Cat owners experienced less deterioration in Short Recall (*p* = 0.042, ES = 0.18) and Long Recall (*p* = 0.014, ES = 0.22) than non-owners. The trajectories of changes with aging were generally in the same directions for dog owners and cat owners, with dog and cat owners experiencing less deterioration in cognitive function than non-owners.

A combined analysis including both dog ownership and cat ownership as independent predictors allowed for simultaneous comparisons with individuals who owned neither cats nor dogs. This adjusted analysis yielded results like those for the separate analyses (Supplemental Table 1).

### Index visit pet ownership and changes in cognitive function (sensitivity analysis)

The relationship of pet ownership at index visit to changes in cognitive function with aging produced generally similar results to those obtained with pet ownership at the time of each cognitive assessment used as the independent variable in the analysis. The relationships of index visit cat ownership and dog ownership to changes in cognitive function produced different results (Supplemental Table 2). The differences in trajectories of changes in cognitive function, according to pet ownership status after controlling for age and co-morbidities were like those reported in Table 4. Taking the pet ownership results together, pet owners exhibited less deterioration in cognitive function than non-owners. However, examining the independent relationships of cat ownership and dog ownership to changes in cognition did not show an association of either dog or cat ownership with changes in cognitive function.

### Differences between dog owners exclusively and cat owners exclusively in Changes in cognitive function

**Figure 2:**
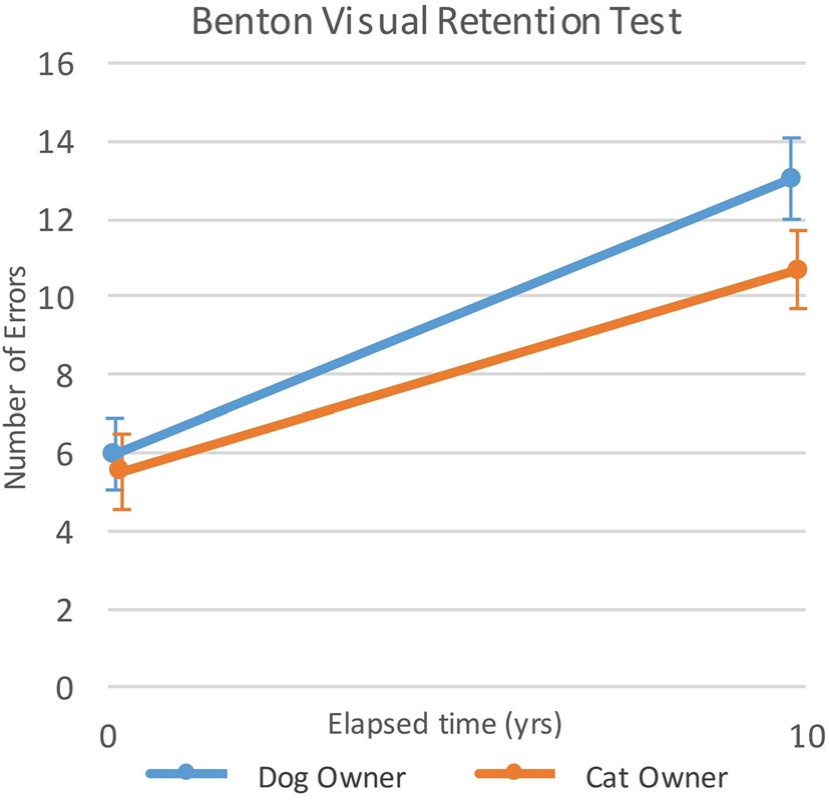
Comparison of changes in number of errors on the Benton Visual Retention Test with aging: comparison of dog owners with cat owners.

Comparison of changes in cognitive function of dog owners exclusively (own only dogs) and cat owners exclusively (own only cats) with aging adjusted for age and comorbidities (Table 5) demonstrated only one significant difference between the groups. Dog owners exclusively had greater deterioration (increase in errors) with aging in Visual Recall than cat owners exclusively (*p* = 0.007, ES = 0.43; Fig. 2).

**Table 5:** Estimates for interaction parameters from linear mixed models a) comparing changes in cognitive function outcomes between dog owners exclusively (who do not own cats) and cat owners exclusively (who do not own dogs) with aging, adjusted for age and comorbidity (n = 141) and b) examining the contributions of dog walking status to changes in cognitive function variables with aging, adjusted for age and comorbidity (n = 73).

Outcome	Cats vs dogs with years of aging			Dog walking vs non-walking with years of aging		
	Estimate	se	p	Estimate	se	*p*
Immediate recall	0.067	0.143	0.468	0.238	0.247	0.334
Short recall	− 0.027	0.045	0.542	0.182	0.075	**0.015**
Long recall	− 0.030	0.040	0.460	0.027	0.071	0.703
Visual recall	0.188	0.069	**0.007**	0.085	0.120	0.479
Trails A(wln)	− 0.003	0.004	0.493	0.010	0.007	0.156
Trails B(ln)	− 0.003	0.005	0.585	− 0.034	0.010	**0.001**
Trails B-A(wln)	− 0.001	0.005	0.802	− 0.032	0.008	** < 0.001**
Digits forward	− 0.008	0.019	0.686	− 0.025	0.034	0.471
Digits backward	0.020	0.021	0.342	0.031	0.356	0.385
Naming(rln)*	0.0003	0.003	0.920	− 0.002	0.004	0.670
Digit symbol	0.103	0.110	0.348	0.054	0.192	0.780

Reference categories are cat ownership, and dog owners who do not walk their dogs.

ln: natural log transformed; rln: reflected then natural log transformed; wln: Winsorized then natural log transformed; Immediate Recall: California Verbal Learning Test-Immediate Recall; Short Recall: California Verbal Learning Test-short delay free recall; Long Recall: California Verbal Learning Test-long delay free recall; Visual Recall: Benton Visual Retention Test; Trails A: Trail Making Test-A; Trails B: Trail Making Test-B; Trails B-A : Trail Making Test B-Trail Making Test A; Digits Forward: WAIS-R Digits Span Test-maximum Digits Forward; Digits Backward: WAIS-R Digits Span Test-maximum Digits Backward; Digit Symbol: WAIS-R Digit Substitution Test; Naming: Boston Naming Test Score.

*Naming was reflected so higher scores indicate worse function.

That bold indicates statistical significance p < .05 is appropriate.

### Dog walking and changes in cognitive function

More than half of dog owners indicated they walked their dogs. At the index visit demographic characteristics and number of comorbidities of the dog owners who walked their dogs and those who did not did not differ (Table 6). Dog owners who walked their dogs performed better on the Trails A Test (*p* = 0.027, ES = 0.29) in unadjusted analysis (Table 6) than those who did not.

**Table 6:** Demographic, pet ownership, and cognitive function characteristics of dog owners comparing those that walk their dogs to those that don’t walk their dogs (n = 73).

Characteristic	N or M	(%) or (SD)	N or M	(%) or (SD)	N or M	(%) or (SD)	Value	*p*
Age in years (yrs.), M (SD)^3^	64.48	(9.89)	64.11	(9.36)	65.92	(12.00)	0.63	0.532
Black, N (%)^2^	12	(16.44)	10	(17.24)	2	(13.33)		1.000
Female, N (%)1	38	(52.05)	27	(46.55)	11	(73.33)	3.43	0.064
< College graduate, N (%)^2^	3	(4.17)	1	(1.75)	2	(13.33)		0.541
Married or partnered, N (%)^2^	55	(76.39)	41	(71.93)	14	(93.33)		0.100
Lives alone, N (%)^2^	14	(19.72)	10	(17.86)	4	(26.67)		0.475
Single family housing, N (%)^2^	66	(91.67)	53	(92.98)	13	(86.67)		0.598
Family income exceeds 50 K, N (%)^2^	62	(87.32)	48	(84.21)	14	(100)		0.189
Currently works, N (%)^1^	47	(65.28)	38	(66.67)	9	(60.00)	0.23	0.629
MMSE, M (SD)^3^	28.63	(2.07)	28.60	(2.35)	28.43	(1.27)	0.18	0.855
Comorbidities, M (SD)^4^	0.68	(1.17)	0.50	(0.80)	1.40	(1.92)		0.175
Follow-up yrs., M (SD)^3^	5.46	(4.31)	5.44	(4.41)	5.51	(4.06)	0.06	0.955
Immediate recall, M (SD)^3^	53.07	(10.96)	53.93	(9.92)	49.71	(14.29)	− 1.29	0.201
Short recall, M (SD)^3^	10.48	(3.40)	10.60	(3.36)	10.00	(3.64)	− 0.59	0.560
Long recall, M (SD)^3^	11.19	(3.53)	11.35	(3.46)	10.57	(3.88)	− 0.73	0.468
Visual recall, M (SD)^3^	6.68	(4.86)	6.39	(4.44)	7.79	(6.28)	0.96	0.341
Trails A(wln), M (SD)^3^	3.31	(0.34)	3.27	(0.32)	3.48	(0.37)	2.26	**0.027**
Trails B(ln), M (SD)^3^	4.26	(0.42)	4.23	(0.40)	4.40	(0.48)	1.45	0.152
Trails B-A(wln), M (SD)^3^	4.51	(0.30)	4.49	(0.28)	4.57	(0.35)	0.91	0.368
Digits forward, M (SD)^3^	6.87	(1.28)	6.89	(1.26)	6.77	(1.42)	− 0.31	0.761
Digits backward, M (SD)^3^	5.34	(1.44)	5.36	(1.44)	5.23	(1.48)	− 0.3	0.768
Naming(rln)*, M (SD)^3^	2.72	(0.25)	2.70	(0.24)	2.79	(0.26)	1.09	0.281
Digit symbol, M (SD)^3^	46.43	(11.09)	48.891	(11.28)	43.64	(11.27)	− 1.56	0.135

ln: natural log transformed; rln: reflected then natural log transformed; wln: Winsorized then natural log transformed; Immediate Recall: California Verbal Learning Test -Immediate Recall; Short Recall: California Verbal Learning Test-short delay free recall; Long Recall: California Verbal Learning Test-long delay free recall; Visual Recall: Benton Visual Retention Test; Trails A: Trail Making Test-A; Trails B: Trail Making Test-B; Trails B-A : Trail Making Test B-Trail Making Test A; Digits Forward: WAIS-R Digits Span Test-maximum Digits Forward; Digits Backward: WAIS-R Digits Span Test-maximum Digits Backward; Digit Symbol: WAIS-R Digit Substitution Test; Naming: Boston Naming Test Score.

^1^Chi-Square test; ^2^Fischer’s exact test; ^3^Student’s t-test; ^4^Wilcoxon rank sum test.

*Naming was reflected so higher scores indicate worse function.

That bold indicates statistical significance p < .05 is appropriate.

**Figure 3:**
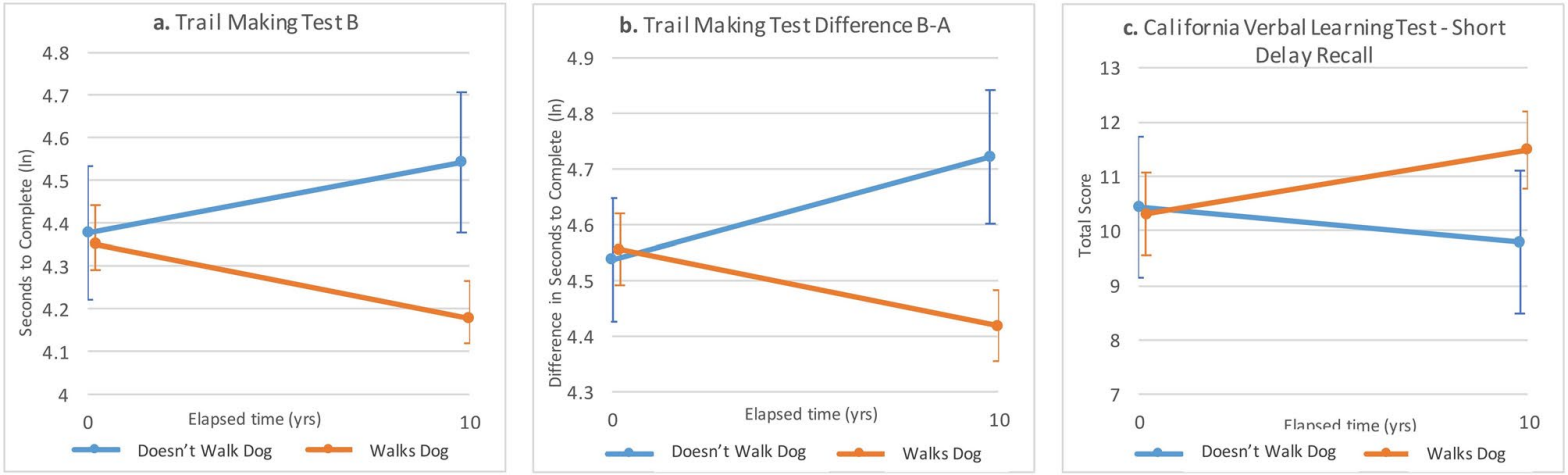
Comparison of changes in selected measures of cognition with aging: comparison of dog owners who do and do not walk their dogs. Note (ln) in axis label indicates a natural log transformation was applied.

Dog walking was related to changes in cognitive function with aging controlling for age and comorbidities (Table 5). Dog owners who walked their dogs experienced significantly different trajectories of changes in Short Recall (*p* = 0.015, ES = 0.54), Trails B (*p* < 0.001, ES = − 0.81) and Trails B-A (*p* < 0.001, ES = − 1.07) than those who did not. Cognitive function improved for dog walkers and deteriorated for non-walkers (Fig. 3).

### Cognitive status, pet ownership and changes in cognitive function

In the current study the participants were generally cognitively functional. All except 41 of the 637 participants (93.6%) were deemed to be cognitively intact, six of the participants (0.94%) were determined to have dementia, 30 (4.7%) to have mild cognitive impairment, and five (7.8%) to have potential cognitive impairment due to strokes or other causes. At the index visit in unadjusted analyses, pet ownership, dog ownership, and cat ownership did not differ significantly between participants who were cognitively intact and those who were not (cognitively intact pet owners = 30.6%, not cognitively intact pet owners = 17.6%, χ^2^ = 3.33, *p* = 0.068; cognitively intact dog owners = 12.8%, not cognitively intact dog owners = 9.8%, χ^2^ = 0.313, *p* = 0.576; cognitively intact cat owners = 12.9%, not cognitively intact cat owners = 2.4%, χ^2^ = 3.51, *p* = 0.061). In LMMs controlling for age and comorbidities, cognitive impairment did not moderate the relationships between pet ownership and changes in most of the cognitive function outcomes, the exception being Long Recall. Long Recall improved among pet owners who were not cognitively intact but not in other subgroups.

## Discussion

In this study using data from the Baltimore Longitudinal Study of Aging, pet ownership, dog ownership, and cat ownership and dog walking were related to the maintenance of executive function and language function, and memory, except working memory, in generally health community-residing older adults as they aged. The use of measures of specific cognitive domains enables examination of the relationship of pet ownership to changes in specific types of cognition. This contrasts with a longitudinal study of community-residing older adults in which cat ownership was not related to the development of the dichotomous outcome of developing mild cognitive impairment after adjustment for potential confounders^26^. In addition, the current study explores changes specific to dog ownership and cat ownership as well as to overall pet ownership.

In the current study, pet ownership, dog ownership, and dog walking were related to slower deterioration in all large domains and all subdomains of executive function except working memory. All three Trails measures revealed reduced deterioration in executive function for pet owners; two of these measures (Trails A, Trails B) also deteriorated more slowly for dog owners than non-owners. Psychomotor speed (Trails tests) and processing speed (Digit Symbol) deteriorated more slowly for pet owners and dog owners than non-owners.

Further, pet ownership, dog ownership, and cat ownership were related to slower deterioration in language function. Only cat ownership was related to slower deterioration in verbal memory.

One mechanism for the slower deterioration in cognition among pet owners, dog owners, and dog walkers is reduction in stress when an animal is present. Evidence supports a relationship of pet ownership with stress biomarkers and psychological perceptions of stress. Chronic stress contributes to cognitive decline in older adults^46^. Elevations of cortisol due to disruptions in the HPA axis may impinge on hippocampal function associated with cognitive function^47–49^.

The presence of pets is well known to decrease stress in experimental studies using both stress biomarkers^19,48,50–52^ and subjective scales^53–55^. Most of these studies documented the positive influence of dogs, but do not include cats. Evidence for pets reducing stress biomarkers in older adults during their normal daily lives is derived from ecological momentary assessment, of blood pressure in adults with pre-hypertension to mild hypertension. Pet owners’ blood pressures in their homes were lower when their dogs were present than when they weren’t present. Cats’ presence was associated with higher systolic as well as lower diastolic blood pressures^56^. These differences in stress biomarkers may be related to differences in interaction of cat owners and dog owners with their respective pets and relate to the observed differences in the deterioration in executive function between dog and cat owners as they age.

Two mechanisms, increased exercise and increased social interaction or social support, could explain the relationship of dog walking to slower deterioration in cognition with aging. Previous research showed that within the biological realm increased physical activity improved cognitive function or decreased deterioration in cognitive function^31,32^. Walking a dog also affects the social realm by increasing social interactions of the dog walkers^57–59^ and improves community social capital^60^.

Differences in deterioration in executive function according to pet and dog ownership and dog walking suggest changes in processing and psychomotor speed. Dog owners may use these skills more frequently to care for pet dogs. Monitoring dog behavior both within the home and on walks, and reacting quickly to environmental concerns (e.g., another dog approaching, a delivery at the door) or other sudden movements all require greater physical and executive skills than are needed to provide homes to more independent cats. Perhaps pet owners need to think and act quickly to care for their pets or prevent their pets from being injured. This may be more important for dog than cat owners, doing this repeatedly could lead to less deterioration with aging.

In the current study, there was no evidence that pet ownership, dog ownership, or cat ownership was related to deterioration in working memory (Digits Forward, Digits Backward). These results contrast with evidence from an experimental study where the presence of a dog was associated with better memory performance in preschool children^61^.

Within the psychological realm, aging leads to reductions in the ability to attend to stimuli and capacity to inhibit irrelevant stimuli^62^. Deterioration in attention (Trails A) was slower among pet owners and dog owners than non-owners, but not related to cat ownership. The tasks required to care for a pet may force older adults to attend to some, while inhibiting other stimuli thus providing additional use and practice in this cognitive domain. Attention switching requires that you quickly shift your focus between multiple processes, another function that may be used more in activities related to caring for a dog or dog walking than to caring for a cat. In general, caring for a pet mandates the ability to direct one’s focus externally and thereby limits the time available for cognitively draining perseveration, worry and anxiety, or rumination. Dog ownership may require a higher level of practice and implementation of goal-directed behaviors and attention shifting consistent with slowing the deterioration of executive function.

In the current study pet ownership, dog ownership, and cat ownership were related to slower deterioration in long-term and verbal memory. Long-term memory is a vast storehouse of information that a person may retain over extended periods of time and has been conceptualized as a permanent repository^63^. The verbal component of this storehouse includes words, labels, and sounds associated with verbal information, whereas the nonverbal component stores information such as images and spatial relationships. All measures of long-term memory (Naming, Short Recall, Long Recall) and most measures of verbal memory (Naming, Immediate Recall, Short Recall, Long Recall) deteriorated more slowly over the ten-year period for pet owners, dog owners, and cat owners. While no studies addressed the relationship of pet ownership to memory, the relationship of chronic stress to poor memory is well established^64^. Furthermore increased social interactions with other humans which occur when individuals have pets may help maintain memory by using it to remember people and their animals.

In the current study, non-verbal memory, assessed with Visual Recall, did not change differently with aging according to pet ownership or cat ownership. Dog ownership was associated with faster deterioration in non-verbal memory. Non-verbal memory deteriorated faster among dog owners than cat owners, suggesting that some aspects of non-verbal memory may be related to cat ownership specifically. The games people play with their cat may require more verbal memory than activities with a dog.

In the current study the measure of language function, Naming, deteriorated more slowly for pet owners, dog owners and cat owners than non-owners with aging. It is likely that language function is used specifically in pet ownership-related tasks, so keeping pets of all kinds confers an advantage. Lower stress and more opportunities for social interaction may support language function similarly to the way they support executive function.

In the current study dog walking was associated with less deterioration in the psychological realm variables of executive function, specifically short-term recall, and psychomotor speed. Dog walking was not associated with changes in other aspects of executive function or language function.

Our findings complement the changes in the social realm demonstrating that dog walking in the community was associated with less loneliness during the COVID-19 pandemic for socially isolated older adults^65^.

In our previous analysis of BLSA physical function data, dog walking was not associated with reduced deterioration in physical function^66^. The physical exercise associated with dog-walking is not a likely explanation for the observed differences in deterioration of cognition with aging among pet owners.

In the current study, moderation analyses did not demonstrate an association of cognitive impairment with the relationship of pet ownership to deterioration in cognitive function with aging. However, almost all the participants were cognitively intact. By reducing stress, pet ownership may minimize deterioration in cognition, more for those who are mildly cognitively impaired than those who are not. Higher chronic stress was associated with faster cognitive decline in individuals with moderate cognitive impairment but not in cognitively normal participants over 3 years^64^. People with worse cognitive function may have already relinquished their pets. However, most of the participants in the BLSA are high functioning and have few comorbidities suggesting an ability to care for pets. Similarly, those who are most frail may have been forced to give up their pets due to living restrictions. However, the relationship of pet ownership to reduced aging-related deterioration was consistent whether pet ownership was categorized at the beginning of the ten years or at the time of each cognitive assessment. One would expect a substantial reduction in pet ownership if deterioration in cognitive function led to discontinuation of pet ownership. This expectation was not achieved; more people obtained a pet (N = 43) than discontinued pet ownership (N = 30) during the follow-up considered in our analysis.

It is important to note that the current study examines the relationship of pet ownership to longitudinal changes in cognitive function in community-residing older adults as they age. This is distinct from therapeutic changes in cognition that might occur with interventions in care homes or other venues. Our findings do not include the presence of the pet during the assessment or an evaluation of how the relationship with the pet may influence the relationships we found.

### Limitations

It is important to note that the current study was conducted on a select group of aging adults. While the study included a relatively balanced sample of men and women, the high socio-economic status, high proportion of majority ethnic/racial groups, and high cognitive function limits the generalizability of the findings to other groups. This also prevents in depth analysis of the role of social determinants of health. Further the large percent of individuals who live with others may not represent the overall older adult population. The generalizability of the negative findings with respect to differences in trajectories of change between dog and cat owners also is limited by the small sample sizes. The contributions of other pet species could not be evaluated due to the small number of individuals who owned pets other than cats or dogs. It is possible that pet ownership will be differentially associated with maintenance of cognitive function according to where the individual lies on the continuum of cognitive function/impairment. While moderation analysis provided little evidence supporting the relationship of cognitive impairment to the association of pet ownership with changes in cognitive function outcomes over time, this is worth further exploration in a more varied population. This study does not investigate whether any of the nuances of pet ownership including pet attachment and pet health or other owner participant characteristics such as marital status or living alone are related to changes in cognitive function, although both being married and not living alone are more common for pet owners than non-owners.

## Conclusion

The current study provides important longitudinal evidence for the contribution of pet ownership to the maintenance of cognitive function in generally health community-residing older adults as they age. Older adult pet owners experienced less decline in cognitive function as they aged, after considering both their pre-existing health and age. Memory, executive function, language function, psychomotor speed, and processing speed deteriorated less over ten years among pet owners than among non-owners and among dog owners than non-owners. Cat owners experienced less deterioration in memory and language function. Dog walking also was associated with slower deterioration in cognitive function. Explanations for the effects reported include decreased stress, increased relaxation/affiliation, increasing external focus for attention, and inhibition of irrelevant thoughts; definitive answers require additional investigation.

This study provides the first longitudinal evidence relating pet ownership and dog walking to reduced deterioration in cognitive function with aging for generally healthy older adults residing in community settings. Policy makers can use these findings to support inclusion of pets in care plans, designing housing and neighborhoods for seniors that are friendly for dog walking^67–69^ and developing programs to support pet ownership and care for older adults’ pets while they are temporarily unable to do so^13^.

### Supplementary Information

Supplementary Tables.

## Data Availability

The datasets generated for this study will not be made publicly available. The study is ongoing, and the data are the property of the National Institutes on Aging. Baltimore Longitudinal Study of Aging data are available through an application process available through their website (https://www.blsa.nih.gov/how-apply).
