# Supplementary material for: Pet ownership and maintenance of cognitive function in community-residing older adults: evidence from the Baltimore Longitudinal Study of Aging (BLSA)
Source: Sci Rep. 2023 Sep 7;13:14738. doi: 10.1038/s41598-023-41813-y (PMC10484936; doi:10.1038/s41598-023-41813-y)
Supplement: Supplementary file 1 — Supplementary Tables. [file 41598_2023_41813_MOESM1_ESM.pdf]

Pet Ownership and Maintenance of Cognitive Function in Community-Residing Older Adults: Evidence from the Baltimore Longitudinal Study of Aging (BLSA)

Erika Friedmann[1], PhD*; Nancy R. Gee[2], PhD; Eleanor M. Simonsick[3], PhD; Melissa H. Kitner-Triolo[3], PhD; Barbara Resnick[1], RN, PhD; Ikmat Adesanya[1], RN, MPH, Lincy Koodaly[1], RN, CRNA; Merve Gurlu[1], MS

1 Department of Organizational Systems and Adult Health, University of Maryland School of Nursing, Baltimore, MD, United States,

2 Center for Human Animal Interaction, Department of Psychiatry, School of Medicine, Virginia Commonwealth University, Richmond, VA, United States,

3 Intramural Research Program, National Institute on Aging, National Institutes of Health, Baltimore, MD, United States

Corresponding Author: Erika Friedmann, PhD, Professor, University of Maryland School of Nursing 655 W. Lombard St., Suite 402, Baltimore, MD 21201
friedmann@umaryland.edu

Supplemental Table 1 Unconditional intraclass correlation coefficients (ICC) to assess dependence for cognitive function variables

Outcome	ICC
**Immediate Recall**	0.77
**Short Recall**	0.73
**Long Recall**	0.74
**Visual Recall**	0.62
**Trails A(wln)**	0.68
**Trails B(ln)**	0.72
**Trails B-A(wln)**	0.51
**Digits Forward**	0.63
**Digits Backward**	0.61
**Naming(rln)***	0.88
**Digit Symbol**	0.80

ln=natural log transformed, rln=reflected then natural log transformed, wln=Winsorized then natural log transformed, Immediate Recall=California Verbal Learning Test -Immediate Recall, Short Recall =California Verbal Learning Test- short delay free recall, Long Recall=California Verbal Learning Test- long delay free recall, Visual Recall=Benton Visual Retention Test, Trails A=Trail Making Test-A, Trails B=Trail Making Test-B, Trails B-A =Trail Making Test B-Trail Making Test A, Digits Forward=WAIS-R Digits Span Test- maximum Digits Forward, Digits Backward=WAIS-R Digits Span Test- maximum Digits Backward, Digit Symbol=WAIS-R Digit Substitution Test, Naming=Boston Naming Test Score; * Naming was reflected so higher scores indicate worse function

Supplemental Table 2. Estimates for interaction parameters from linear mixed models examining the independent contributions of dog ownership and cat ownership to changes in cognitive function variables with aging, adjusted for age and comorbidity (n=146)

	Dog Ownership Interaction with Years of Aging			Cat Ownership Interaction with Years of Aging		
**Outcome**	**Est**	**se**	***p***	**Est**	**se**	***p***
**Immediate Recall**	0.226	0.102	**0.027**	0.233	0.0998	**0.020**
**Short Recall**	0.053	0.031	0.083	0.061	0.030	**0.042**
**Long Recall**	0.040	0.030	0.182	0.071	0.029	**0.015**
**Visual Recall**	0.103	0.047	**0.027**	-0.061	0.045	0.175
**Trails A(wln)**	-0.006	0.003	0.052	-0.004	0.003	0.192
**Trails B(ln)**	-0.010	0.004	**0.010**	-0.005	0.004	0.153
**Trails B-A(wln)**	-0.008	0.004	**0.043**	-0.001	0.004	0.763
**Digits Forward**	-0.005	0.013	0.728	0.002	0.013	0.876
**Digits Backward**	0.018	0.014	0.193	-0.011	0.013	0.413
**Naming(rln)***	-0.004	0.002	**0.033**	-0.005	0.002	**0.015**

*non-ownership reference category,
ln=natural log transformed, rln=reflected then natural log transformed, wln=Winsorized then natural log transformed, Immediate Recall=California Verbal Learning Test -Immediate Recall, Short Recall =California Verbal Learning Test- short delay free recall, Long Recall=California Verbal Learning Test- long delay free recall, Visual Recall=Benton Visual Retention Test, Trails A=Trail Making Test-A, Trails B=Trail Making Test-B, Trails B-A =Trail Making Test B-Trail Making Test A, Digits Forward=WAIS-R Digits Span Test- maximum Digits Forward, Digits Backward=WAIS-R Digits Span Test- maximum Digits Backward, Digit Symbol=WAIS-R Digit Substitution Test, Naming=Boston Naming Test Score; * Naming was reflected so higher scores indicate worse function

Supplemental Table 3. Estimates for interaction parameters from linear mixed models examining the contributions of Pet Ownership, dog ownership, and cat ownership at the index visit to changes in cognitive function variables with aging, adjusted for age and comorbidity (n=637)

	Pet Ownership Interaction with Years of Aging			Dog Ownership Interaction with Years of Aging			Cat Ownership Interaction with Years of Aging		
**Outcome**	**Est**	**se**	***p***	**Est**	**se**	***p***	**Est**	**se**	***p***
**Immediate Recall**	0.294	0.069	**<0.001**	0.105	0.101	0.301	0.285	0.105	**0.007**
**Short Recall**	0.052	0.021	**0.013**	0.005	0.031	0.865	0.051	0.032	0.112
**Long Recall**	0.050	0.020	**0.013**	-0.009	0.030	0.774	0.081	0.031	**0.009**
**Visual Recall**	0.017	0.031	0.587	0.146	0.046	**0.002**	-0.092	0.047	0.051
**Trails A(wln)**	-0.009	0.002	**<0.001**	-0.001	0.003	0.675	-0.003	0.003	0.321
**Trails B(ln)**	-0.009	0.003	**0.001**	-0.004	0.004	0.291	-0.008	0.004	**0.043**
**Trails B-A(wln)**	-0.004	0.003	0.089	-0.002	0.004	0.612	-0.006	0.004	0.115
**Digits Forward**	0.008	0.009	0.363	0.001	0.013	0.958	-0.002	0.014	0.865
**Digits Backward**	0.009	0.009	0.314	0.009	0.014	0.513	0.013	0.014	0.370
**Naming(rln)***	-0.005	0.001	**<0.001**	-0.002	0.002	0.375	-0.004	0.002	0.073
**Trails A(wln)**	-0.009	0.002	**<0.001**	-0.001	0.003	0.675	-0.003	0.003	0.321
**Trails B(ln)**	-0.009	0.003	**0.001**	-0.004	0.004	0.291	-0.008	0.004	**0.043**
**Trails B-A(wln)**	-0.004	0.003	0.089	-0.002	0.004	0.612	-0.006	0.004	0.115
**Digits Forward**	0.008	0.009	0.363	0.001	0.013	0.958	-0.002	0.014	0.865
**Digits Backward**	0.009	0.009	0.314	0.009	0.014	0.513	0.013	0.014	0.370
**Digit Symbol**	0.050	0.049	0.302	-0.102	0.072	0.160	0.075	0.074	0.306

Non-ownership is the reference category, ln=natural log transformed, rln=reflected then natural log transformed, wln=Winsorized then natural log transformed, Immediate Recall=California Verbal Learning Test -Immediate Recall, Short Recall =California Verbal Learning Test- short delay free recall, Long Recall=California Verbal Learning Test- long delay free recall, Visual Recall=Benton Visual Retention Test, Trails A=Trail Making Test-A, Trails B=Trail Making Test-B, Trails B-A =Trail Making Test B-Trail Making Test A, Digits Forward=WAIS-R Digits Span Test- maximum Digits Forward, Digits Backward=WAIS-R Digits Span Test- maximum Digits Backward, Digit Symbol=WAIS-R Digit Substitution Test, Naming=Boston Naming Test Score; * Naming was reflected so higher scores indicate worse function
